# Some Queer Symptoms of Lyssa

**Published:** 1891-06

**Authors:** 

**Affiliations:** Meran


### SOME QUEER SYMPTOMS OF LYSSA.

DR. PROELL, MERAN.

In a village a boy was suddenly taken down with lyssa and died, though there was no mad dog all around. A few days before the boys played soldiers, and one of them, who personated the commander, went up into an attic and got an old, rusty sword. Accidentally he slightly wounded the boy and the lyssa followed. An old man recollected that many years ago there was a mad dog in the village which was probably killed by the same sword, and then, without cleaning it, it was thrown among old lumber up into the attic. Probably a minute part of the poison clung to it, and it did not lose its virulence for years, though it became very rusty. Proell was (1848) interne at the surgical wards of the Vienna Hospital, when a patient was brought in who constantly laughed and complained of unbearable itching. He was immediately put in a separate room and two internes were ordered to remain with him and watch him. In spite of excruciating pains he was patient and often begged us, when giving him medicine, to be careful that he may not injure us. He dictated us a farewell letter to his family, and with the Lord's prayer on his lips he passed away. Nearly the same sorrowful resignation was observed in another patient who also died in less than forty-eight hours after being bitten. A young lady had a pet dog, and, from mere mischief her lover threw it on her bare neck, which frightened her greatly, so that she uttered a scream sounding like the barking of a dog. Many years have passed, and still the woman utters the same bark in stores and people have become used to it. Another consequence of her fright was, she could not withhold her thoughts; she had to talk them out, though they might have been offensive, for it was really an *incontinentia idearum involuntaria*. S. L.

Allg. Hom. Zeit. 6, '91.

